# Loss of *hif-1* promotes resistance to the exogenous mitochondrial stressor ethidium bromide in *Caenorhabditis elegans*

**DOI:** 10.1186/s12860-016-0112-x

**Published:** 2016-09-13

**Authors:** Muntasir Kamal, Dayana R. D’Amora, Terrance J. Kubiseski

**Affiliations:** 1 Department of Biology, York University, Toronto, Canada; 2 Department of Neuroscience, York University, Toronto, Canada; 3 Present address: Department of Molecular Genetics, University of Toronto, Toronto, Canada

**Keywords:** *C. elegans*, DJ-1, Hypoxia inducible factor, Mitochondria, p38 MAPK

## Abstract

**Background:**

Mitochondrial dysfunction is one of the leading causes of neurological disorders in humans. Mitochondrial perturbations lead to adaptive mechanisms that include HIF-1 stabilization, though the consequences of increased levels of HIF-1 following mitochondrial stress remain poorly understood.

**Results:**

Using *Caenorhabditis elegans*, we show that a *hif-1* loss-of-function mutation confers resistance towards the mitochondrial toxin ethidium bromide (EtBr) and suppresses EtBr-induced production of ROS. In mammals, the PD-related gene DJ-1 is known to act as a redox sensor to confer protection against antioxidants and mitochondrial inhibitors. A deletion mutant of the *C. elegans* homolog *djr-1.1* also showed increased resistance to EtBr. Furthermore, our data implicates p38 MAP kinase as an indispensable factor for survival against mitochondrial stress in both *hif-1* and *djr-1.1* mutants.

**Conclusions:**

We propose that EtBr-induced HIF-1 activates pathways that are antagonistic in conferring protection against EtBr toxicity and that blocking HIF-1 activity may promote survival in cells with compromised mitochondrial function.

## Background

Cells depend on mitochondria to generate the energy required to carry out vital cellular processes. Specific mutations and environmental toxins that target mitochondria are responsible for many degenerative disorders in humans, such as Parkinson’s disease (PD), dementia, Alzheimer’s disease and mitochondrial myopathy [1]. Mitochondrial dysfunction usually occurs as a result of the organelle’s failure to handle stress resulting from normal processes of energy production, due to the loss of one or more mitochondrial protein subunits via deletions or point mutations of mitochondrial DNA (mtDNA), mutations in nuclear DNA that encodes mitochondrial proteins, or the loss of protective mechanisms that ensure maintenance of a viable environment to allow these essential processes to occur [2, 3].

Loss of mitochondrial electron transport chain (ETC) subunits or mtDNA results in increased ROS production, and this activates antioxidant defense systems. Interestingly, the transcription factor hypoxia inducible factor-1 (HIF-1), which is usually stabilized during hypoxic conditions (conditions of low oxygen concentration) as a protective mechanism, is also stabilized in *C. elegans* with perturbed mitochondria [4]. In mammals, HIF-1 in hypoxic situations plays an important role in hypoxia resistance by continuing to provide the cell with energy from sources that do not require oxygen [5]. Not surprisingly, many tumours exhibit hypoxic environments and thus rely on functional HIF-1 to survive [6]. Importantly, this HIF-1-dependent pathway is favored if mitochondrial function is blocked, since energy production will then depend on anaerobic pathways. A hallmark of many cancer cells is mitochondrial dysfunction accompanied by a hyperactive HIF-1 [7]. Unfortunately, the role of HIF-1 in the pathogenesis of mitochondrial diseases is much less understood, partly due to the fact that severe mitochondrial perturbations readily lead to apoptosis in vitro, making long-term studies difficult. Furthermore, most HIF-1 research focuses on mitochondrial perturbation predominantly in the context of tumourigenesis, rather than on mitochondrial diseases themselves.

In this study, we used *C. elegans* to examine the role of HIF-1 on the protective responses activated when mitochondrial function is impaired and report that in contrast to wild type animals, *hif-1* loss-of-function mutants exhibited high resistance to the mitochondrial toxin Ethidium Bromide (EtBr). Since mammalian HIF-1 was shown previously to be downstream of the PD associated gene product DJ-1 [8], we tested a strain containing a deletion of the nematode ortholog *djr-1.1* and found that it was also resistant to EtBr. Furthermore, the *C. elegans* p38/MAPK pathway was strongly induced in EtBr, and was indispensable for growth in both *hif-1* and *djr-1.1* mutants, indicating that this pathway is involved in protective changes upon mitochondrial perturbations. Taken together, our study shows a role for *C. elegans hif-1* in regulating growth and survival during mitochondrial perturbation, and that the *C. elegans* p38/MAPK cascade is an essential component of a protective response pathway that is activated when mitochondrial function is compromised.

## Results

### *hif-1* and *djr-1.1* mutants are resistant to EtBr exposure compared to wild type worms

The nematode *C. elegans* undergoes a series of larval developmental stages. The transition from L3 to L4 is associated with a five-fold increase in mtDNA content, and this increases an additional six-fold in transitioning from L4 to adult, as a result of germline expansion [9]. Various studies have shown that one of the effects of mitochondrial perturbation on the health of worms is the reduction of brood size, or fecundity [9–11]. Furthermore, in mammalian cells, it has been shown that EtBr intercalates between the base pairs of double-stranded DNA and inhibits transcription and replication [12, 13]. The effect of EtBr is mostly impairment of mitochondrial DNA and EtBr is used to rid cells of mitochondrial DNA to create ρ^o^ cells [14]. Furthermore, in *C. elegans*, EtBr has been shown to activate the mtUPR as evidenced by the increase expression of *hsp-60*, the mitochondrial chaperone that responds to mitochondrial dysfunction upon EtBr exposure [15, 16]. Although it is possible that EtBr has some effect on nuclear DNA, it is negligible compared to its effect on the mitochondria [17].

**Fig. 1:**
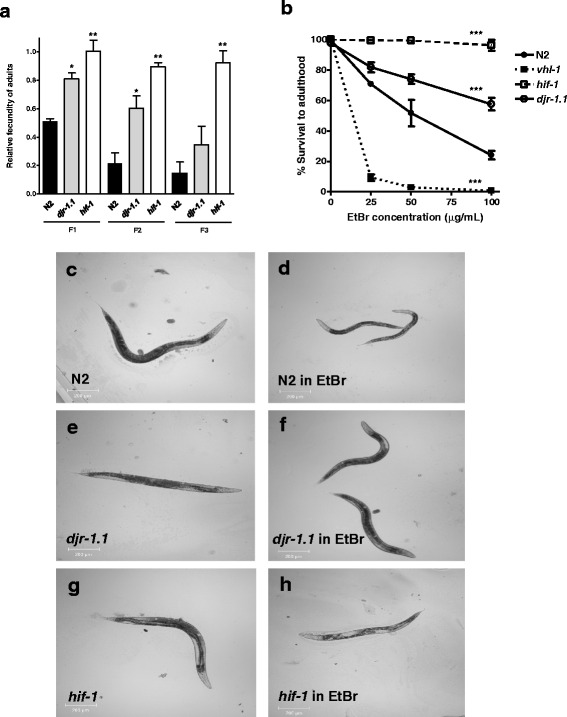
*hif-1* and *djr-1.1* mutants are highly resistant to EtBr. **a** *hif-1* animals exposed to EtBr have no effect on fecundity over three generations, while wild type worms showed a significant decrease in brood size. Brood size of N2 (wild type), *djr-1.1* and *hif-1* animals in 25 μg/mL EtBr were counted for three generations: F1, F2 and F3. Relative fecundity is measured as the number of progeny grown to adulthood on EtBr divided by the number of progeny grown to adulthood in the absence of EtBr, and would include animals that arrest at the L3 stage. Animals with ruptured vulva were not counted. Asterisk marks denote significant difference of fecundity between P0 (normalized to 1.0) and the following generations for each strain (* denotes *p* < 0.05 and ** *p* < 0.01) using one-way Anova. **b** Concentration dependence of EtBr. N2, *hif-1, vhl-1* and *djr-1.1* mutant animals (1st generation) were grown on worm plates containing nematode growth media and various concentrations of EtBr (0, 25, 50 and 100 μg/mL). The number of worms that grew to adulthood was determined and plotted. To determine significant differences between strains, the data were subjected to two-way ANOVA comparing groups (different strains) over different concentrations of EtBr. All strains showed a significant difference compared to N2 (***p < 0.001). **c**-**h** Growth of strains on EtBr. N2, *djr-1.1* and *hif-1* worms (as labeled) four days after egg-laying in plates containing either no EtBr (**c**, **e**, **g**) or 50 μg/mL EtBr (**d**, **f**, **h**)

In order to test the roles of *hif-1* and *djr-1.1* in maintaining the health of worms in which mitochondrial function is impaired by EtBr, we compared the brood sizes of the loss-of-function mutants *hif-1* and *djr-1.1* to that of the wild type (N2) grown on EtBr plates. We initially determined the brood size of these strains under normal conditions, and found that *hif-1* mutant animals had a lower brood size than N2 (Table 1). In contrast, wild type worms grown on plates containing 25 μg/mL EtBr had a greatly reduced fecundity over three generations (F1, F2 and F3) compared to both *djr-1.1* and *hif-1*mutants. Indeed, *hif-1* mutant animals showed to be significantly resilient over three generations as there was no effect on the fecundity of this strain (Fig. 1a). Demands for reproduction are believed to compromise the worm's ability to resist certain stresses (e.g., hypoxia) [4]. This result shows that increased EtBr resistance is not a consequence of reduced brood size.

**Table 1:** Brood size of N2 (wild type) and mutant animals

Strain	Brood size (no. of eggs ± S.D.)
N2	229.0 ± 23.8
*hif-1(ia4)*	149.3 ± 9.8*
*djr-1.1(tm918)*	263.0 ± 24.1

Single worms were grown in fresh OP50 plates at 22 °C, and the adult worm was transferred to a fresh plate containing OP50 everyday until the worm stopped laying eggs. Figures are mean ± standard deviation (S.D.). Results are from three independent experiments (*n* ≥ 40). One-way Anova (*denotes *p* < 0.05) was done to test for statistical significance in brood size between mutant strains and wild-type using GraphPad Prism 5.0 software

Since the brood size of *djr-1.1* and *hif-1* mutant animals were less affected by EtBr, we tested if the growth of these animals was different from that of wild type worms with EtBr exposure. Previous studies have reported that exposing embryos to EtBr causes L3/L4 larval arrest, indicating that lower mtDNA content cannot support transition to advanced developmental stages that accompany germline proliferation [9, 18, 19]. Here, we found that whereas nearly 50 % of wild type animals showed L3 larval arrest on 50 μg/ml EtBr, only ~25 % *djr-1.1* mutants arrested at the L3 stage while almost 100 % of *hif-1* mutants were able to reach adulthood (Fig. 1b-h). At higher EtBr doses, *hif-1* mutants retained the high EtBr resistance while *djr-1.1* mutants exhibited moderate resistance to EtBr (Fig. 1b-h). These results indicate that the loss of *djr-1.1* and particularly *hif-1* confers resistance in worms against EtBr-induced mitochondrial perturbation.

**Fig. 2:**
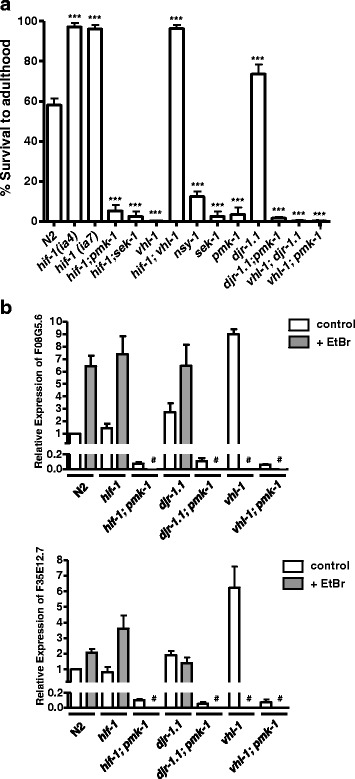
*hif-1* and *djr-1.1* mutants require the *nsy-1*/*sek-1*/*pmk-1* signalling pathway for EtBr resistance. **a** *pmk-1* is required by *hif-1* and *djr-1.1* for growth on EtBr containing plates. The first generation of various worm strains containing single or double mutant alleles as listed were grown on worm plates containing nematode growth medium and 50 μg/ml of EtBr, and the percentage of animals that reached the adult stage were determined. Two single mutants of *hif-1*, *hif-1(ia4)* and *hif-1(ia7)*, demonstrated growth in the presence of EtBr. All subsequent experiments were done with the *hif-1(ia4)* allele. Furthermore, it was found that the loss of *pmk-1* abrogates EtBr resistance of *hif-1* and *djr-1.1* mutants. Asterisk marks denote significant difference between EtBr-treated N2 and EtBr-treated mutants (* denotes *p* < 0.05 ** *p* < 0.01, *** *p* < 0.001). One-way Anova was performed to test for the statistical significant difference in growth between strains. **b** *pmk-1* is induced in the presence of EtBr in wild type, *hif-1, djr-1.1* and *vhl-1* mutants. Transcript levels of two *pmk-1* targets (*F08G5.6* and *F35E12.5*) in control (0 μg/mL, “control” open box) and EtBr-treated (50 μg/mL, “ + EtBr”; grey box) samples were measured using qRT-PCR. # indicates not determined, as *vhl-1* mutant and *pmk-1* double mutants were unable to survive on worm plates containing EtBr. Results are from three independent experiments

To further our understanding for the basis of *hif-1* and *djr-1.1* mutants ability to survive in the presence of EtBr, we tested a number of other strains for their ability to grow on worm plates containing 50 μg/ml of EtBr (Fig. 2a). One of the other strains we tested was the *hif-1(ia7)* mutant and found that it displayed resistance to EtBr, indicating that the phenotype demonstrated with the *hif-1(ia4)* mutant in Fig. 1 was not due to a background mutation. VHL-1 is the worm ortholog of the mammalian Von-Hippel Lindau tumour suppressor [20] and loss-of-function *vhl-1* stabilizes HIF-1 [21]. We found that the *vhl-1* mutant strain demonstrated a very marked sensitivity to EtBr (Fig. 1b and 2a) probably due to the absence of VHL-1 resulting in HIF-1 stabilization [21]. Furthermore, we found that the *hif-1;vhl-1* double mutant exhibits a similar level of tolerance to EtBr to that of the *hif-1* single mutant (Fig. 2a).

*p38 MAPK is required for survival on EtBr -*MAP kinases are activated when mitochondrial function is impaired and are believed to play a protective role in stressed organisms [22–24]. The *C. elegans* stress-activated kinase PMK-1 is involved in innate immunity and provides protection to bacterial pathogens and oxidative stress [25–27]. Therefore, we asked if the p38/PMK-1 pathway was required for survival on EtBr. Indeed, *nsy-1*, *sek-1* and *pmk-1* mutants were all extremely sensitive to EtBr, with almost 80 % and close to 100 % of the population undergoing larval arrest for *nsy-1* and *sek-1* and *pmk-1*, respectively (Fig. 2a). Importantly, *pmk-1* was also required for the increased resistance of *djr-1.1* and *hif-1* animals, with *hif-1* also requiring *sek-1* for its resistance (the effect of *sek-1* mutation on *djr-1.1* was not tested): *djr-1.1; pmk-1*, *hif-1;pmk-1* and *hif-1;sek-1* double mutants were all highly susceptible to EtBr, similar to *pmk-1* and *sek-1* single mutants. Although the requirement of *nsy-1* for *djr-1.1* and *hif-1* phenotypes was not tested, our data suggest that the *nsy-1/sek-1/pmk-1* cascade is required for protection against EtBr toxicity.

Having demonstrated that *pmk-1* was required for EtBr resistance, we asked whether *pmk-1* was activated upon EtBr exposure. We tested this by quantifying the expression of two genes, *F08G5.6* and *F35E12.5*, previously shown to be targets of *pmk-1* but with no clear function [26, 28]. We reproduced the earlier finding that loss of *djr-1.1* results in higher *pmk-1* activation (i.e., overexpression of these genes) under normal conditions [26] (Fig. 2b). Notably, *pmk-1* was strongly activated in both wild type and *djr-1.1* and *hif-1* mutant animals in EtBr, except for *F35E12.5* in *djr-1.1* animals; this suggests that the already high expression of this gene in the *djr-1.1* mutant is sufficient to respond to EtBr. In general, the EtBr-induced *pmk-1*-activation in these mutants was similar in magnitude to that of wild type worms (Fig. 2b). As a control, we generated double mutants of *pmk-1* with *hif-1* and *djr-1.1*, resulting in a complete inability of the two reporter genes to express in the presence or absence of EtBr, demonstrating that the expression of the reporter genes were dependent on PMK-1 activity (Fig. 2b). Surprisingly, *vhl-1* mutant worms also demonstrated higher PMK-1 activity, at least with respect to the genes tested (Fig. 2b). Therefore, although higher PMK-1 activity is required, enhanced PMK-1 activity itself is not sufficient for growth in the presence of EtBr.

### ROS levels are lower in *hif-1* than in wild type nematodes exposed to EtBr

**Fig. 3:**
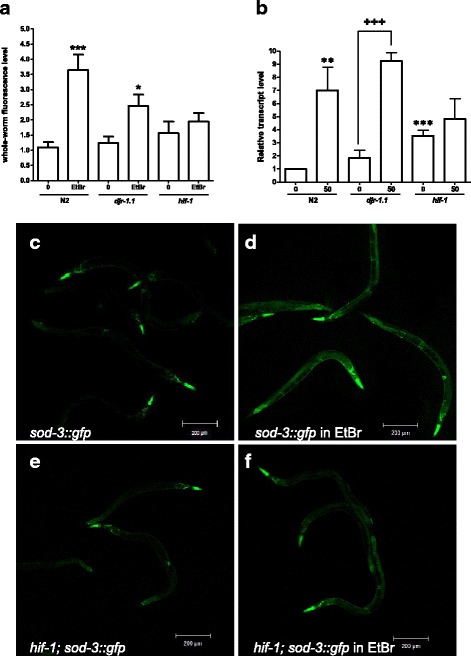
ROS production is enhanced in EtBr-treated wild type and *djr-1.1* worms, but not *hif-1* mutant worms. **a** In vivo ROS levels were determined in L4/adult worms grown on worm plates containing either EtBr (50 μg/mL) or water. Worms were subsequently incubated in buffer containing H_2_DCF-DA and whole organism fluorescence intensity measured using a Zeiss LSM 700 confocal microscope. Results were summarized from at least 4 independent experiments. Fluorescence intensity was quantified using ImageJ software. The y-axis depicts absolute values of mean intensity per worm. Statistical analysis was performed in GraphPad Prism 5.0. Asterisk marks denote significant difference between control and drug-treated worm samples for each strain (* *p* < 0.05, ** *p* < 0.01, *** *p* < 0.001). **b** *sod-3* is overexpressed in EtBr-treated wild type and *djr-1.1* animals but not EtBr-treated *hif-1* animals. mRNA levels of *sod-3* was determined by qRT-PCR in wild type, *hif-1* and *djr-1.1* mutants grown on worm plates containing nematode growth media in either the absence (labeled 0) or presence (labeled 50) of 50 μg/ml of EtBr, using *act-1* as a control. Asterisk/plus signs indicate statistical significant difference in expression between control N2 sample and other strains and that between untreated and EtBr-treated sample for each strain (*/+ denotes *p* < 0.05, **/++ *p* < 0.01, ***/+++ *p* < 0.001). *sod-3* transcript levels were significantly higher in wild type and *djr-1.1* animals, but not *hif-1* mutants. **c** Wild type *sod-3::gfp* and *hif-1;sod-3::gfp* strains adults were placed on plates containing EtBr and images were taken of five to 10 worms three and four days post egg-laying for *hif-1;sod-3::gfp* and wild type animals, respectively

Previous studies have indicated that mitochondrial perturbation results in elevated ROS levels in the mitochondria, which might further compromise the organelle’s ability to handle ROS [29–31]. To measure in vivo ROS levels, we used the compound H_2_DCF-DA (2′, 7′-dihydrochlorofluorescein diacetate) [29]. We suspended L4/adult worms treated with either EtBr or water in M9 buffer containing H_2_DCF-DA and measured whole organism fluorescence intensity by confocal microscopy. We found that wild type and, to a lesser extent, *djr-1.1* worms, had elevated ROS levels, while the EtBr-treated *hif-1* animals showed no significant increase (Fig. 3a). This indicates that the presence of a functional *hif-1* gene and protein leads to higher endogenous ROS levels upon EtBr treatment.

The gene encoding the mitochondrial superoxide dismutase, *sod-3* is overexpressed in EtBr-treated animals indicating elevated ROS levels in these animals [31]. Interestingly, it was reported that DAF-16 translocates to the nucleus when *hif-1* is knocked down by RNAi [32]. Since *sod-3* is a well-known target for DAF-16 [33], we tested if *sod-3* transcript levels differed in the mutant worms from that of wild type following EtBr exposure. Under normal conditions, *sod-3* transcript levels were slightly higher in *hif-1* animals, while *sod-3* levels in *djr-1.1* and wild type were similar (Fig. 3b). EtBr exposure resulted in *sod-3* overexpression in wild type and *djr-1.1* worms, but we found no significant change in expression in EtBr-treated *hif-1* samples. This was confirmed by increased *sod-3::gfp* expression in EtBr-treated wild type animals but no increase in *sod-3::gfp* expression in *hif-1* animals (Fig. 3c-f). Taken together, our data suggest that ROS levels and hence, oxidative stress, is lower in EtBr-treated *hif-1* animals.

### The *hif-1*-dependent gene tyrosinase tyr-2/TRP2 is overexpressed in EtBr

**Fig. 4:**
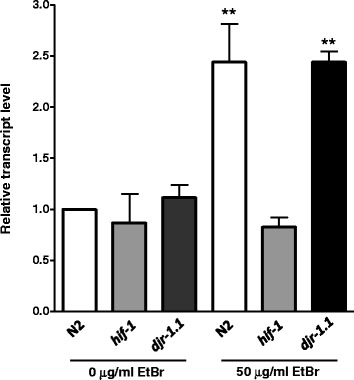
The *hif-1*-dependent gene *tyr-2* is overexpressed in wild type and *djr-1.1* mutants but not in *hif-1* animals treated with EtBr. The transcript level of *tyr-2* was determined by qRT-PCR in wild type, *hif-1* and *djr-1.1* mutant worms grown on worm plates in the absence and presence of 50 μg/ml of EtBr. The experiment was performed independently at least three times. GraphPad Prism 5.0 was used to find statistical significance. Asterisk marks denote statistical significant differences (* denotes *p* < 0.05, ** *p* < 0.01, *** *p* < 0.001) in *tyr-2* expression in wild type and *djr-1.1* between untreated and EtBr treated samples but not in *hif-1* animals

Previously, it was shown that EtBr activates the expression of well-established *hif-1* dependent genes such as *nhr-57* and *cysl-2* [11]. To confirm that in our system we were seeing an increase in the expression of *hif-1* dependent genes upon exposure to EtBr, we looked at the expression of *tyr-2*. The *C. elegans tyr-2* gene was first reported to be overexpressed with hypoxia treatment (0.5 % oxygen) in a *hif-1*-dependent manner [34], and was later shown to be responsible for the anti-apoptotic property of HIF-1 by suppressing *cep-1/p53*-dependent germline apoptosis following ionizing radiation [21]. *hif-1* mutants exhibit very low *tyr-2* mRNA transcript levels, while *vhl-1* mutants show higher *tyr-2* transcript levels, which is consistent with the idea that *tyr-2* regulation is *hif-1* dependent [21]. Here, we found that *tyr-2* was overexpressed in EtBr-treated samples, showing an almost two-fold increase in EtBr treated wild type and *djr-1.1* samples that did not differ between the two strains (Fig. 4). As expected, *tyr-2* expression did not increase in EtBr-treated *hif-1* mutant samples (Fig. 4). These results confirm the previous report [11] that HIF-1 gene expression activity occurs in the presence of EtBr in wild type worms.

## Discussion

This study was carried out to investigate the role of HIF-1 in survival of worms subjected to mitochondrial stress. Previous results have indicated that overexpression of HIF-1 in *C. elegans* promotes longevity in a manner parallel to SKN-1/NRF and DAF-16/FOXO transcription factors [35]. Here, we found that *hif-1* mutants grew significantly better on EtBr compared to wild type worms, indicating that the *hif-1*-dependent response activated in EtBr antagonizes any adaptive mechanism required for EtBr tolerance. Furthermore, our results show for the first time to our knowledge that the p38 MAPK pathway is strongly induced in worms with compromised mitochondrial function, and that *pmk-1* is indispensable for EtBr survival. Finally, we also show that ROS levels are lower in *hif-1* worms, indicating that p38/PMK-1 activity and lower oxidative stress in *hif-1* mutant animals probably both contribute to promote survival in the presence of EtBr.

In *C. elegans*, inactivation of genes encoding subunits of the mitochondrial respiratory chain can stabilize HIF-1, and a *hif-1*-dependent hypoxia response is induced in animals with compromised mitochondrial function [36]. Because mitochondrial ROS generation is increased in these animals and because mitochondrial ROS stabilizes HIF-1 during hypoxia, a hypoxia response may be initiated during mitochondrial stress that may not necessarily be specific to hypoxia but modified to act as a survival pathway specific to mitochondrial stress. Alternatively, it is possible that *hif-1*-induced pathways may have pleiotropic effects on survival, as a consequence of hypoxia-specific pathways being altered during mitochondrial stress when not accompanied by true hypoxic condition. We did not test if a full *hif-1* dependent hypoxia response or at least specific genes in the hypoxia pathway which might determine the EtBr phenotype were induced; thus, it will be interesting to test if mutations in *hif-1*-dependent hypoxia signalling pathways confer worms with a similar resistance towards EtBr.

Our findings add to the growing body of evidence that suggests the consequence of HIF-1 stabilization will depend on the type and/or severity of perturbation: for example, with mitochondrial stress produced in ETC. point mutants [36] or diluted ETC (by RNAi knockdown) animals [37], HIF-1 may have a chiefly beneficial or a protective function and is required for lifespan extension. But in other types of stress, such as from an exogenous stressor like EtBr, the same or additional HIF-1-independent pathways are activated that reduce mitochondrial stress. In this case, HIF-1 activation is likely to be “reactive” in nature, and may activate pathways that are superfluous, or even antagonistic to mitochondrial-stress specific pathways. We propose that such inappropriate effects of HIF-1 activation are a direct consequence of its complex, pleiotropic role on cellular stress response in general.

In mammals, the gene *DJ-1* encodes a protein that can act as a redox-sensor, and can act as an antioxidant during oxidative stress and is upstream of HIF-1 [8]. *C. elegans* has two homologues, *djr-1.1* and *djr-1.2*. Surprisingly, we found that *djr-1.1* mutants were significantly more resistant to EtBr than wild type worms, a phenotype it shares with *hif-1*, indicating that these two genes probably share overlapping functions. The NSY-1/SEK-1/PMK-1 pathway confers innate immunity to worms against bacterial pathogens, and *djr-1.1* mutation potentiates this signalling [26]. Therefore, activation of immune signalling via NSY-1/SEK-1/PMK-1 may increase the survival of worms against various stresses including perturbation of mitochondrial function and *djr-1.1* worms probably had greater survival in EtBr because of higher *pmk-1* activation under normal conditions. However, since *djr-1.1* mutants were susceptible to a higher EtBr dose, it is conceivable that the limit of *pmk-1* activation in these animals restricts their ability to resist EtBr toxicity.

Our results have implicated the p38 MAPK pathway as an important factor in regulating survival against mitochondrial stress. In agreement with our finding, Liu et al. [38] reported worms interpret mitochondrial perturbation as a xenobiotic/pathogen infection, eliciting a xenobiotic and pathogenic response. Since the ASK-1/MKK3/6/p38 MAPK pathway is specifically responsible for triggering apoptotic cell death and neuronal loss in PD, it will be important to precisely identify downstream targets of p38 MAPK that are activated upon EtBr exposure that are essential for survival in EtBr, so that pharmacological intervention to enhance the activity of those genes can slow down or improve pathogenesis of PD and other mitochondrial diseases.

## Conclusions

We propose that HIF-1 activates pathways upon exposure to the mitochondrial disrupter EtBr that hinders survival of cells experiencing this type of mitochondrial stress. Many tumour tissues exhibit pathological hypoxic conditions and have a hyperactive HIF-1 [39]. Tumour cells also tend to have impaired mitochondrial function, such that HIF-1 makes the appropriate metabolic shift to anaerobic respiration that ultimately contributes to the cancer phenotype [40]. Consequently, current cancer treatments target HIF-1 in order to force cancer cells to depend on the dysfunctional mitochondria, in the hope that cancer cells would then fail to grow and ultimately be out-competed by normal cells. We propose the possibility that HIF-1-dependent gene expression might hinder survival in cells experiencing mitochondrial stress so that loss of HIF-1, especially in normoxic cancer cells, might actually be a benefit to cells. However, since worms do not have a HIF-2 homologue, the role of HIF-1 in worms may differ in significant ways from that in more complex organisms. Therefore, it will be important to validate the findings of this study in other model organisms.

## Methods

### *C. elegans* strains

Worm strains were provided by either the Caenorhabditis Genetics Center (CGC, University of Minnesota, Twin Cities, USA) or by the Mitani Lab through the National Bio-Resource Project of the MEXT, Japan. All the worms were outcrossed a sufficient number of times prior to order to ensure a uniform genetic background. All double mutants were created using standard *C. elegans* techniques and confirmed by single worm PCR to identify deletions when possible. Primers used for genotyping and qRT-PCR are available upon request. The N2 (Bristol) is the wild type worm strain, and other strains used were: AU3 [*nsy-1(ag3)*], CF1553 [muIs84*(sod-3::gfp)*], KU4 [*sek-1(km4)*], KU25 [*pmk-1(km25)*], YF134 [*hif-1(ia4); sek-1(km4)*], YF136 [*djr-1.1(tm918)*], YF137 [*hif-1(ia4); vhl-1(ok161)*], YF138 [*djr-1.1(tm918); pmk-1(km25)*], YF143 [*hif-1(ia4);* muIs84*(sod-3::gfp)*], YF149 [*hif-1(ia4)*; *pmk-1(km25)*], YF154 [*vhl-1(ok161)*], YF179 [*djr-1.1(tm918); vhl-1(ok161)*], YF180 [*djr-1.1(tm918); pmk-1(km25)*], YF190 [*vhl-1(ok161); pmk-1(km25)*], ZG31 [*hif-1(ia4)*] and ZG596[*hif-1(ia7)*].

### Single-worm PCR (SW-PCR)

Individual worms were placed in PCR tubes containing 4 μL 1× Thermopol® reaction buffer (NEB) and 0.5 mg/mL proteinase K buffer (NEB) and incubated at −80 °C for 30 min, then incubated at 65 °C for 1 h and 95 °C for 15 min to inactivate proteinase K. SW-PCR was initiated by adding 20 μL of PCR mix that contained primer pairs, dNTPs, and 1× Thermopol® reaction buffer for a reaction volume of 24 μL. The PCR cycle consisted of the following steps: 55 °C for 60 s, 94 °C for 5 min 15 s, 55 °C for 15 s, 72 °C for 1 min (35 cycles), and 72 °C for 5 min (total reaction time 1 h and 30 min).

### Brood size assay in control and EtBr plates at 22 °C

Single worms were grown in fresh OP50 plates, and the adult worm was transferred to a fresh plate containing OP50 every day until the worm stopped laying eggs. Total number of eggs laid was recorded by counting number of L1 larvae and unhatched eggs. Brood size of N2 (wild type), *djr-1.1* and *hif-1* animals at 22 °C in 25 μg/mL EtBr for three generations F1, F2 and F3 were recorded. For each generation, single worms were grown in fresh OP50 plates containing 25 μg/mL EtBr or double-distilled water (control), and the adult worm was transferred to a fresh plate containing 25 μg/mL EtBr or distilled water seeded with 50 μL OP50 every day until the worm stopped laying eggs. For each strain, relative fecundity is defined as the ratio of total number of eggs laid in EtBr plates to total number of eggs laid in control (no EtBr) plates.

### EtBr treatment and survival assay

To make 25, 50 and 100 μg/mL EtBr plates, 10 mg/mL original stock EtBr was diluted with double-distilled water to 0.5, 1.0 and 2.0 mg/mL, respectively. 250 μL of diluted EtBr was added to worm plates for desired EtBr concentration. The plates were left to dry for two days, after which they were seeded with 50 μL OP50. For survival assays, only 50 and 100 μg/mL EtBr were used. Six gravid adults were placed on each of two plates (i.e., duplicates) containing 50 or 100 μg/mL ethidium bromide (EtBr) seeded with 50 μL OP50 bacteria and allowed to lay eggs for three hours (Day 1). On Day 2, total number of L1 animals in the two plates after egg-hatching were counted. Between Days 4–6, total number of adults in the two plates were counted and recorded as a percentage of total number of L1’s observed on Day 2. The experiment was done three to seven times, with the number of L1’s per plate greater than 40. For each strain, experiments were carried out to record proportion of L1’s reaching adulthood in control plates; it was observed that >99 % of population grew to adulthood in control plates for all strains studied, and thus control plates were not included in the EtBr survival experiments.

### Quantitative reverse-transcriptase PCR (qRT-PCR)

Total RNA was isolated using the standard Trizol method, using manufacturer’s instructions. For the PCR step, master mix was prepared with forward and reverse primers (0.5 μg/μL) diluted 1/10 in a final volume 40 μL. They were further diluted by adding 3.7 μL of the diluted primers to 25.3 μL of RT-PCR grade water for each strain (total volume 29 μL). To this was added 4 μL of cDNA, followed by addition of 33 μL SYBR® Advantage® qPCR Premix (Clontech). *act-1* was used as an internal control; 20 μL of samples were loaded in 96-well plates in triplicates, and performed with comparative C_T_ (∆∆C_T_) as the quantification method with the following steps: 50 °C for 20 s and 95 °C for 10 s (holding stage), 95 °C for 15 s and 60 °C for 1 min (cycling stage, 40 cycles), 95 °C for 15 s, 60 °C for 1 min, 95 °C for 30 s and 60 °C for 15 s (melt curve stage).

### ROS quantification

For each strain, four gravid adults were allowed to lay eggs for 6 h in plates containing 50 μg/mL EtBr and seeded with 50 μL OP50 bacteria (Day 1). On Day 4, L4 or adult worms were washed with M9 buffer and incubated in 1 mL M9 containing 10 μM H_2_DCF-DA (dissolved in DMSO) for 45 min in the dark. After incubation, the tubes were spun at a low speed (2,000 rpm) and worms were placed on unseeded agar plates. Live worms were then placed on slides with an agarose pad, covered with a cover-slip, and, using confocal microscopy, z-stack analysis was performed with the same exposure, magnification (20×) and number of slices (ten) for all individual worms and strains. Alexa Fluor 488 was used as the emission spectrum. Five to ten worms were analyzed for each strain per experiment. Fluorescence intensity was quantified using ImageJ software.

### DIC microscopy

Live worms were placed in a drop of 2 mM levamisole on a slide with an agarose pad and closed with a cover-slip. All images were taken using a confocal microscope at 20× magnification, with default DIC settings.

## Abbreviations

DIC: Differential interference contrast
EtBr: Ethidium bromide
ETC: Electron transport chain
H_2_DCF-DA: 2′,7′-dihydrochlorofluorescein diacetate
HIF-1: Hypoxia inducible factor 1
mtDNA: Mitochondrial DNA
mtUPR: Mitochondrial unfolded protein response
PCR: Polymerase chain reaction
PD: Parkinson’s disease
qRT-PCR: Quantitative reverse transcriptase polymerase chain reaction
ROS: Reactive oxygen species
